# Learning and forgetting in the jet fighter aircraft industry

**DOI:** 10.1371/journal.pone.0185364

**Published:** 2017-09-28

**Authors:** Anelí Bongers

**Affiliations:** Department of Economics and Economic History, Universidad de Málaga, Málaga, Spain; Universidad de Castilla-La Mancha, SPAIN

## Abstract

A recent strategy carried out by the aircraft industry to reduce the total cost of the new generation fighters has consisted in the development of a single airframe with different technical and operational specifications. This strategy has been designed to reduce costs in the Research, Design and Development phase with the ultimate objective of reducing the final unit price per aircraft. This is the case of the F-35 Lightning II, where three versions, with significant differences among them, are produced simultaneously based on a single airframe. Whereas this strategy seems to be useful to cut down pre-production sunk costs, their effects on production costs remain to be studied. This paper shows that this strategy can imply larger costs in the production phase by reducing learning acquisition and hence, the total effect on the final unit price of the aircraft is indeterminate. Learning curves are estimated based on the flyaway cost for the latest three fighter aircraft models: The A/F-18E/F Super Hornet, the F-22A Raptor, and the F-35A Lightning II. We find that learning rates for the F-35A are significantly lower (an estimated learning rate of around 9%) than for the other two models (around 14%).

## Introduction

The final acquisition cost of a fighter aircraft depends on three main factors. First, procurement price is related to the embodied technology of the equipment. As the technical and performance characteristics of an aircraft increase, the cost of such aircraft will also do so [1]. Second, the unit acquisition cost is related to average fixed costs. Average fixed costs depend on two variables: Total sunk costs and the total number of aircraft manufactured. The production of aircraft implies an important pre-production fixed cost (due to design, experimentation, prototypes, etc.) and thus, fixed average costs can be an important component of the final price. Finally, the unit acquisition cost depends on the intensity of the learning-by-doing process during production, which implies a reduction in average cost as accumulated production increases. Ziemer and Kelly noted that jet aircraft prices are affected by a number of factors, such as the number of units to be ordered, the production rate, the learning curve, or differences in price due to different producers [2].

One well-known key phenomenon in a wide range of industries is the fact that the production cost is a decreasing function of cumulative production. This is a direct consequence that cumulated output turns into experience in production, which leads to higher productivity. Such phenomenon is represented by the so-called learning curve, also known as progress curve. The learning curve was first studied by Wright in the aircraft industry [3]. Wright observed that the number of man-hours inputs needed for the production of an airframe was a decreasing function of the total number of previously produced airframes of the same type. Learning curve models were first used to estimate aircraft production cost prior to World War II (WWII). Later, this model has been applied to a large variety of industries, thus turning into a key instrument for strategic management. Learning or progress functions became highly popular among management consultants and engineers after WWII. This popularity was mainly due to the fact that progress functions were a simple tool very useful to be applied to a complex phenomenon. See, for instance, [4] and [5] for a description of the origins of the learning curve and its applications.

The basic idea behind learning-by-doing is simple. Workers and organizations “learn” to perform a repeated task and, consequently, labour efficiency depends on the number of previously produced units. When decreasing production costs are observed, several factors can explain such phenomenon, being learning by doing one of them. Learning by doing can be interpreted as a sort of dynamic economies of scale. The learning process can appear at an individual worker level (labour learning) or at a plant level (organizational learning), see [6]. One key characteristic of the learning curve is that the rate of learning is greater at the beginning of the production and therefore, it is a decreasing function of the number of units produced. Learning means that potential economies of scale can be exploited, resulting in average or unit cost decreases as the level of output accumulates. In general terms, the learning curve states that as the total quantity of produced units doubles, the cost per unit decreases at a constant rate.

In the literature, we find several alternative approaches to studying the implications of learning-by-doing from a theoretical point of view. A branch of the literature has focused on incorporating the learning by doing process into the neoclassical production cost theory, in order to develop a dynamic cost approach. Seminal papers in this area are [7], [8], and [9], among others. A second branch of the literature studies the implications of learning by doing for industry structure and pricing. Cost reductions due to learning effects can have important implications for market structure and economic welfare, by creating barriers to entry and protecting early entrants from effective market competition. Seminal papers of this branch of the literature are [10], [11], [12], and [13]. In this context, the existence of the learning curve can provide a rationale for a pricing and marketing strategy in which producers initially price low in order to increase sales, thereby quickly accumulating experience and exploiting the cost-reducing effect of production learning. Indeed, this is the basis for the recommendations by the Boston Consulting Group [14]. A concept related to learning by doing is that of organizational forgetting, which implies that the accumulated stock of production experience can depreciate over time. Empirical evidence shows that experience depreciation can be very important in a wide range of industries, not only in the case of interruptions in production but also as a simultaneous process implicit in the learning-by-doing process. Finally, at an aggregate level, learning-by-doing is considered as one of the factors producing endogenous growth [15].

The estimation of learning curves is a key element to determine the pricing of military aircraft. Usually, military equipment procurement processes are not competitive at the production stage, and they are characterized by a monopoly-monopsony market structure, with a single producer and a sigle buyer. On the other hand, learning curves are the fundamentals of government decision-making on how many units of a particular aircraft will be procured. This is particularly important, as the price of fighter aircraft has soared dramatically during the last decades. In this context, a recent strategy pursued by the military aircraft industry has consisted in the development of a single airframe with different technical and operational specifications. This strategy has been designed in order to reduce costs in the Research, Design and Development phase, which are pre-production sunk costs, with the ultimate objective of reducing the final unitary price of the aircraft. This is the case of the F-35 Lightning II, where three versions, A, B, and C, with significant differences among them but based on the same airframe, are produced simultaneously. Whereas this strategy can be useful to reduce pre-production sunk costs, their effects on production costs and on the final price of the aircraft remain to be studied.

This paper contributes to the literature by studying the implications of that new strategy on the dynamic economies of scale associated with learning and forgetting processes. For that purpose, this paper estimates learning curves for the three more recent fighter aircraft models (the F/A-18E/F Super Hornet, the F-22A Raptor and the F-35A Lightning II) using flyaway cost as a proxy for production cost. We find an 85% learning curve for both the Super Hornet and the Raptor. Nevertheless, results for the F-35A show a lower learning rate, with an estimated learning curve of around 90%. Two main conclusions can be drawn from these results. First, the learning by doing process does not change as the technological complexity of airframes increases. Indeed, we find that learning in the production of the F-22A, a fifth generation aircraft, is of a similar magnitude as the one observed in the production of the F/A-18E/F, a fourth generation less technological advanced aircraft. Second, we cast doubts on the strategy of developing a single airframe with different versions to be produced simultaneously on the same assembly line. Whereas this strategy can be useful in reducing fixed costs, our results detect a lower learning process during the production phase, which implies a lower rate of reduction of production costs in the assembly of the aircraft as accumulated production increases. Our results are consistent with the ones found by Kleiner, Nickelsburg and Pilarski, who studied learning and forgetting processes in the production of the DC-9 and MD-80 aircraft which shared the same fuselage, and found that the simultaneous production of both models on the same assembly line provoked a rise in production costs [16]. This could also be the case of the F-35 Lightning II.

The remainder of the paper is structured as follows. Section 2 describes briefly the backgrounds of the learning curve and introduces the concept of organizational forgetting. Section 3 reviews the empirical literature. Data and variables are described in Section 4. Section 5 presents the results and the discussion. Finally, Section 6 presents some conclusions.

## The learning curve

Learning curve models have been developed in order to explain the observed phenomenon of increasing productivity as production accumulates in a variety of industries. Learning curves were developed initially in the seminal paper by Wright based on the observation that the unit labour requirement in airframe manufacturing declined at a constant rate as cumulative output doubles, which implies that the unit cost of production falls at a decreasing rate [3]. The pioneer work of Wright, which developed the so-called cumulative average model, was confirmed by Crawford who developed the so-called “unit” learning model [17]. Learning effects appear mainly in the assembly process, and therefore they are related to the human production factor, not only at individual level but also at the level of the organization [6].

In general, we assume that the output of an industry is produced with the following technology:

$$\begin{array}{c} Y_{t}=A_{t}F\left(K_{t},L_{t}\right) \end{array}$$ (1)

where *Y*_*t*_ is output, *K*_*t*_ is capital inputs and *L*_*t*_ is labour. *F*(⋅) is a production function and *A*_*t*_ is a measure of Total Factor Productivity (multi-factor productivity or neutral technology) defined as:

$$\begin{array}{c} A_{t}=A\left(E_{t},t\right) \end{array}$$ (2)

where *E*_*t*_ represents cumulated experience and *t* is time. That is, learning-by-doing is a component of Total Factor Productivity. Following [18], *A*_*t*_ can be defined as:

$$\begin{array}{c} A_{t}=A_{0}E_{t}^{\beta }e^{\gamma t} \end{array}$$ (3)

where *A*_0_, *β* and *γ* are constants. That is, productivity depends on cumulated experience (interpreted as a type of dynamic returns to scale) and on calendar time, representing the standard measure of exogenous neutral or Hicks technical change. Cumulated experience is proxied by cumulated production.

Given the relationship between productivity and cumulated experience by expression (3), the learning curve can be defined in terms of the following mathematical function:

$$\begin{array}{c} P_{i}=aE_{i}^{\beta } \end{array}$$ (4)

where *P*_*i*_ is the number of labour hours (or the price) required to produce the *i* unit, *a* is the number of labour hours or the cost required to produce the first unit, *E* is the cumulative number of units produced reflecting “experience”, and *β* is the learning index which defines the slope of the learning curve. The basic formulation of the learning curve can be extended by incorporating additional factors into the cumulative experience, such as industry spillovers, production rate, organizational forgetting, etc.

In spite of the widely empirically observed learning effects, this phenomenon has not yet been grounded in any theoretical model, although learning-by-doing has been incorporated into several existing models. From a theoretical point of view, learning curves make reference to a context in which the production process is dynamic. This contrasts with the standard neoclassical cost function theory, which is developed in a static framework and thus does not take into account the acquisition of knowledge through experience. As a consequence, researchers initially tried to incorporate learning-by-doing into the classical production cost theory, using alternative approaches. First extensions were carried out by [7] and [8], who developed a dynamic theory of production based on the progress function, linking learning with the classical static cost theory. Contributions in the same direction are [9], [19], [20] and [21], who incorporate learning processes into the neoclassical production cost theory through the specification of a dynamic cost function, where learning-by-doing can be understood as a kind of dynamic return-to-scale.

An alternative attempt to develop a theoretical framework for learning-by-doing, at a macroeconomic level, was Arrow, which is considered as the pioneering work of endogenous growth literature [15]. Although learning curves consider the cumulative output as the index of experience, [15] used cumulative gross investment as the variable representing experience, which makes it possible for the existence of a continuous learning process. The idea is similar to that of the standard learning curve but replacing cumulative output by cumulative investment as the measure of experience. [22], [23] and [24] extended Arrow’s model. Stokey developed a dynamic general equilibrium model in which goods are valued according to the characteristics they contain and where learning by doing is the force behind sustained growth [25]. Young studied the dynamic effects of international trade under the existence of learning by doing [26].

Finally, another branch of the literature dealing with the effects of learning curves has focused on the study of their strategic implications in industrial organization. In this context, learning processes may have important implications for market structure and economic welfare, since they can create a barrier to entry and reduce market competition. Indeed, one of the main implications of the work of the Boston Consulting Group was the advice to produce a lot at an early stage, in order to move down the learning curve faster than rival firms as a strategy for gaining strategic advantage [14]. Lee studied the implications of learning-by-doing for a competitive firm [10]. Spence shows that learning would raise entry barriers in a dynamic pricing model [11]. Rosen studied the case of a Cournot quantity-setting model showing that learning curves create entry barriers against late entrants [12]. [13], and [27] both conclude that it is better to set a price below marginal cost, particularly at the beginning of the introduction of the new product. More recently, Cabral and Riordan studied the strategic price-setting implications of the learning curve in a differentiated duopoly setting [28].

### Organizational forgetting

Learning-by-doing implies a continuous fall in marginal cost as experience in production is accumulated and hence, it is assumed to be a persistent process over time. Related to the learning-by-doing process is the notion of organizational forgetting. The concept of organizational forgetting was first introduced by Argote, Beckman and Epple [29]. Whereas the standard learning curve approach assumes that learning is cumulative and persistent over time, [29] show that knowledge acquired via learning by doing might depreciate rapidly. Forgetting can only occur in a context of learning. Depreciation of experience can be easily understood in the case of interruptions in production. After a period of interruption, when production is restarted, productivity is observed to be lower than in the previous periods (see, for instance, [30]). This is the case, for instance, of a strike or a stoppage in the production process due to a negative shock on demand or to shortages of assembly parts. Nevertheless, as pointed out by [29], forgetting can occur even when there are no interruptions in production or other factors limiting production but as a process directly related to learning.

Under the organizational forgetting hypothesis, experience, *E*, is assumed to depreciate at a constant exponential rate *δ* and to accumulate as a result of production experience:

$$\begin{array}{ccc} E_{1} & = & 0,\\ E_{i} & = & 1+e^{-\delta t}E_{i-1} \end{array}$$ (5)

where *t* is the calendar time at which unit *i* is produced. Organization forgetting has been incorporated into the definition and the estimation of learning curves. At a theoretical level, [31] extend the learning-by-doing model of [28] by including organizational forgetting. They show that learning by doing and organizational forgetting are empirically important for the industry dynamics and that forgetting can offset the effects of learning by increasing competition.

## Empirical literature

There is a vast empirical literature estimating learning curves in a number of industries. Although the concept of the learning curve was initially developed in the aircraft industry, it has rapidly been extended to other products. Examples are [3], [32], [33], [34], [35], [36], [37], [38], [39], [40], [41] and [42] for the aircraft industry; [43], [44], [19], [29], [45], [46] and [47] for the shipbuilding industry; [48, 49] and [50] for energy power plants; [51] for the automobile industry; [52] and [18] for semiconductors, etc. Empirical studies including organizational forgetting are, for instance, [29], [53], [41], [54], [55] and [56].

On the other hand, learning curves have been widely used as a planning instrument popularized by the Boston Consulting Group [14]. One of the main applications of the learning curve by [14] was the advice to firms to produce as much as possible at an early stage in order to move it down faster than rival firms to gain strategic advantage. Furthermore, the learning-by-doing curve is a planning tool used by the Department of Defense (DoD) in the acquisition process of airplanes.

Perhaps the more well-known empirical application of learning curves has been for the shipbuilding industry, particularly thanks to the Liberty program. The Liberty program was launched by the U.S. Maritime Commission in 1941, calling for a massive expansion of the merchant fleet. A total 2,708 ships were produced under this program. This program meant the largest ever production of a single ship design (a total of 2,580 units of the same vessel-type were produced) in a number of shipyards. Availability of cost and production data from this program and the fact that all ships were of the same design made this case study the most popular in the learning-by-doing empirical literature. The Liberty-type ships were constructed in 16 different yards during WWII. The first study based on this case was carried out by Searle who estimated a model which related man-hours per output with accumulated output, using data from 10 yards [43]. He found that from December 1941 to December 1944, man-hours required to produce a Liberty vessel fell from an index value of 100 to 45, that is, an impressive increase in productivity of 122% during the whole period, approximately 40% per year. Searle estimated that each doubling of cumulative output reduced labour hours per ship by between 12% and 24%. Lane studied productivity growth in the Liberty program at individual yards, arriving at similar conclusions [44].

Rapping estimated a production function in which output (number of ships) depends on labour inputs and physical capital services using data from 15 yards, obtaining the existence of increasing returns to scale [19]. He found that each doubling of accumulated output is accompanied by a 29% increase in output, keeping inputs constant. He also studied output at individual yards, arriving at similar results with an average productivity growth of 23% (between 11% and 34% at individual yards). Argote, Beckman and Epple used the same dataset to study the dynamics of learning and its depreciation [29]. They showed that the measured learning in terms of accumulated output significantly overstates the persistence of learning. Using data from 13 yards, they estimated that from the stock of knowledge at the beginning of a year, only 3.2% would remain one year later, which means that knowledge depreciates rapidly. Nevertheless, Thompson studied the Liberty ships case in the Calship, showing that the increases in output per worker are explained by an increase in capital intensity and a reduction in quality, where other factors as changes in production technology and capacity utilization also play a significant role [46]. Thornton and Thompson focused on the study of knowledge spillover effects among different shipyards, showing that they are an important source of productivity growth, even more than learning itself [45]. Their results contrast with the ones obtained by [29], which found only very limited evidence of spillover effects among the different shipyards involved in the Liberty program.

The other industry which has received special attention by the empirical literature is the aircraft industry. In fact, the learning curve was first applied to aircraft production by Wright, who observed that the number of man-hours inputs needed for the production of an airframe were a decreasing function of the total number of previously produced airframes of the same type [3]. Wright estimated a learning curve of about 80%. Based on these results, learning curves were used by the U.S. Air Force and the industry for estimating the cost of producing airframes, as shown by [32].

Alchian studied direct man-hours in the production of 22 military aircraft, including bombers, fighters, trainers, and transports during WWII, focusing on the prediction error derived from the estimated progress curve [33]. Sturmey estimated learning curves for 18 British aircraft [34], and Reinhardt studied costs and revenues in the Lockheed Tri-Star L-1011 program, estimating a value for the learning curve of 77.4% [37]. Womer and Patterson estimated learning curves for the C-141 cargo aircraft [56]. They use different specifications and find a learning curve of between 76% and 81%. Frischtak computed the learning curve for the Brasilia EMB-120 aircraft produced by Embraer and finds a 72.7%-81.8% curve for the airframe and a 74.7%-82.9% for the fuselage [39]. Mishina studied the Boeing B-17 Flying Fortress program and found a decrease of 27.9% in the number of working hours per machine whenever production doubled, noting that productivity gains were mainly from improvements in the production system rather than in the learning process [40].

Argote, Beckman and Epple also studied the L-1011, focusing on the persistence of learning [29]. They analysed the importance of adjustment costs of the rate of output. In fact, the L-1011 TriStar program was characterized by wide variations in the rate of output over time. Production costs reported by Lockheed seem to suggest that depreciation of learning was an important factor and that costs will rise when the rate of production falls. Benkard estimated several alternative specifications of the learning curve for the Locked L-1011 TriStar aircraft, considering forgetting, adjustment cost, input prices and spillovers [41]. He found a learning rate between 18% and 53%. Benkard estimates a dynamic oligopoly model for the commercial aircraft industry to analyse industry pricing, industry performance, and optimal industry policy, computing a dynamic model for the market of wide-bodied commercial aircraft including the learning curve as a key element [42]. More recently, Kleiner, Nickelsburg and Pilarski studied learning and forgetting processes in the McDonnell-Douglas MD-80 and DC-9, concluding that organizational forgetting is virtually non-existent in this case [16].

Empirical evidence is not restricted to the shipbuilding and aircraft industries, but learning curves have been estimated in a large variety of industries, such as power generation, automobile industry, and semiconductor, among others. Joskow and Rozanski studied the learning process in the construction of nuclear plants and the increase in electricity production as a result of the experience gained in this industry [48]. Zimmerman found that the production cost of nuclear power plants not only decreases as a result of companies increasing their level of knowledge but also because of the experience gained by the industry, again highlighting the importance of spillover effects [50]. Joskow and Rose, on the contrary, found that the transfer of experience among coal generating industries is very limited [49]. Levitt, List and Syverson estudied learning in the automobile industry using highly detailed data from an assembly plant of a major auto producer and found that most of the substantial learning by doing knowledge at the plant was not retained by the plant’s workers, even though they were an important conduit for knowledge acquisition [51]. Irwin and Klenow studied 32 firms from several countries producing different kinds of semiconductor chips, considering also possible spillover effects and obtaining learning rates between 14% and 28.7% [18]. Meanwhile, Gruber studied the learning processes in the industry of semiconductor memory chips, obtaining that learning processes are very different for different chips, although they are very similar [57]. Empirical literature has extended the analysis of learning by including organizational forgetting. Darr, Argote and Epple studied the acquisition, transfer, and depreciation of knowledge in pizza stores. They found that learning by doing depreciates rapidly in these stores [53]. Finally, David and Brachet studied organizational learning and forgetting for a dataset of ambulance companies and found that organizational forgetting is an important phenomenon due to both labour force turnover and skill decay [55].

## Data and variables

The main focus of this paper is to quantify the learning process in the latest fighter aircraft produced by the aerospace industry. In particular, we estimate learning and forgetting processes for three recent fighters: The F/A-18E/F Super Hornet produced by Boeing, and the F-22A Raptor and the F-35A Lightning II, both models produced by Lockheed Martin. The objective is twofold. First, we want to study whether learning processes in the industry have changed in the production of the new more technological advanced aircraft. Particularly, how the learning process of 5th generation fighters differs from that of the 4th generation ones. Second, we are interested in studying the impact on the learning process of the last strategy designed by the industry to reduce the total cost of new generation fighters, by developing a single airframe with different technical and operational specifications.

In the literature, learning curves have been estimated using alternative variables: man-hours, labour cost, production cost, and final price, depending on data availability and industry characteristics. Irwin and Klenow showed that under Cournot competition, there is a strict relationship between prices and marginal costs [18]. In our case, given data limitations, learning curves are estimated using the unit flyaway cost for each aircraft. The flyaway cost measures the cost of production and production tools essential for building a single unit, excluding sunk costs such as research and development, all supplementary costs for supporting the equipment, and future costs such as spares and maintenance. The flyaway cost values the price of an aircraft in terms of its marginal cost, and thus can be considered as a good proxy for the production cost. Furthermore, when pricing an aircraft, the flyaway cost of the 100th unit is commonly used, as indicated by [58] and [2], in order to take into account the learning curve and its effect on the production costs. In the literature, it is assumed that, by the hundredth unit of production of a new fighter aircraft, additional learning is relatively minor [2]. This is because most of the improvement takes place during the early units of production, and assuming an 80% learning, the curve will eventually become almost flat, and thus, the flyaway cost of additional units remains almost constant. As pointed out by Ziemer and Kelly [2], pag. 312, “the price of the hundredth unit, expressed in dollars of the time period when the first production contract is signed, represents the best estimate of the actual resource cost.” Therefore, the estimation of the learning curve is crucial in order to determine the price of military aircraft and, in fact, it is a tool used in the procurement process of this type of equipment.

Notice that the market studied in the paper is not an oligopoly (duopoly), and there is no firms’ market power in this particular market, as there is a sigle producer and a sigle buyer (monopoly-monopsony). Markets for military aircraft are very different from markets for civil aircraft. In the civil aircraft markets firms compete among each other and the final price is probably not a good proxy for production cost. In the particular case of the U.S. aircraft industry, there are only two firms producing fighters: Lockheed Martin and Boeing. However, these two firms do not compete in the production phase. Competition between these two firms occurs only in the pre-production phase. The DoD opens a procurement process subject to a set of technical and performance characteristics, and supplies financial resources to both firms to develop a prototype. Then, the DoD evaluates the performance and technical characteristics of the two prototypes and choose a single winner (that is, only one of the two firms will lead the prototype to the production phase). Once a firm (Lockheed or Boeing) has been selected, there is a negotiation process between the DoD and the winning firm about the number of units to be produced and the procurement priceto be paid for each lot, in which estimation of the learning curve plays a key role. Margins are also fixed in this procurement process.

**Fig 1:**
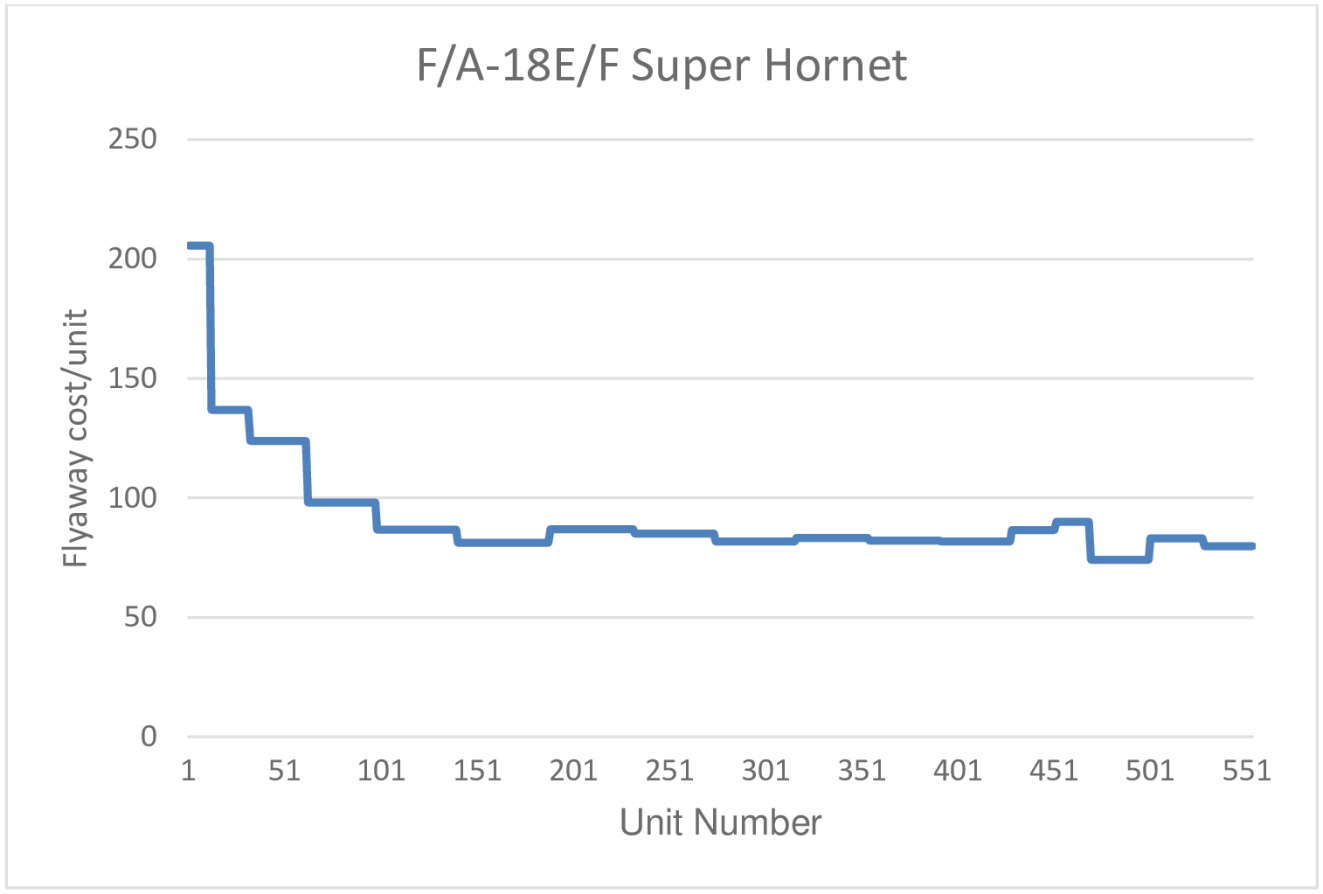
F/A-18E/F Super Hornet. Unit flyaway cost.

**Fig 2:**
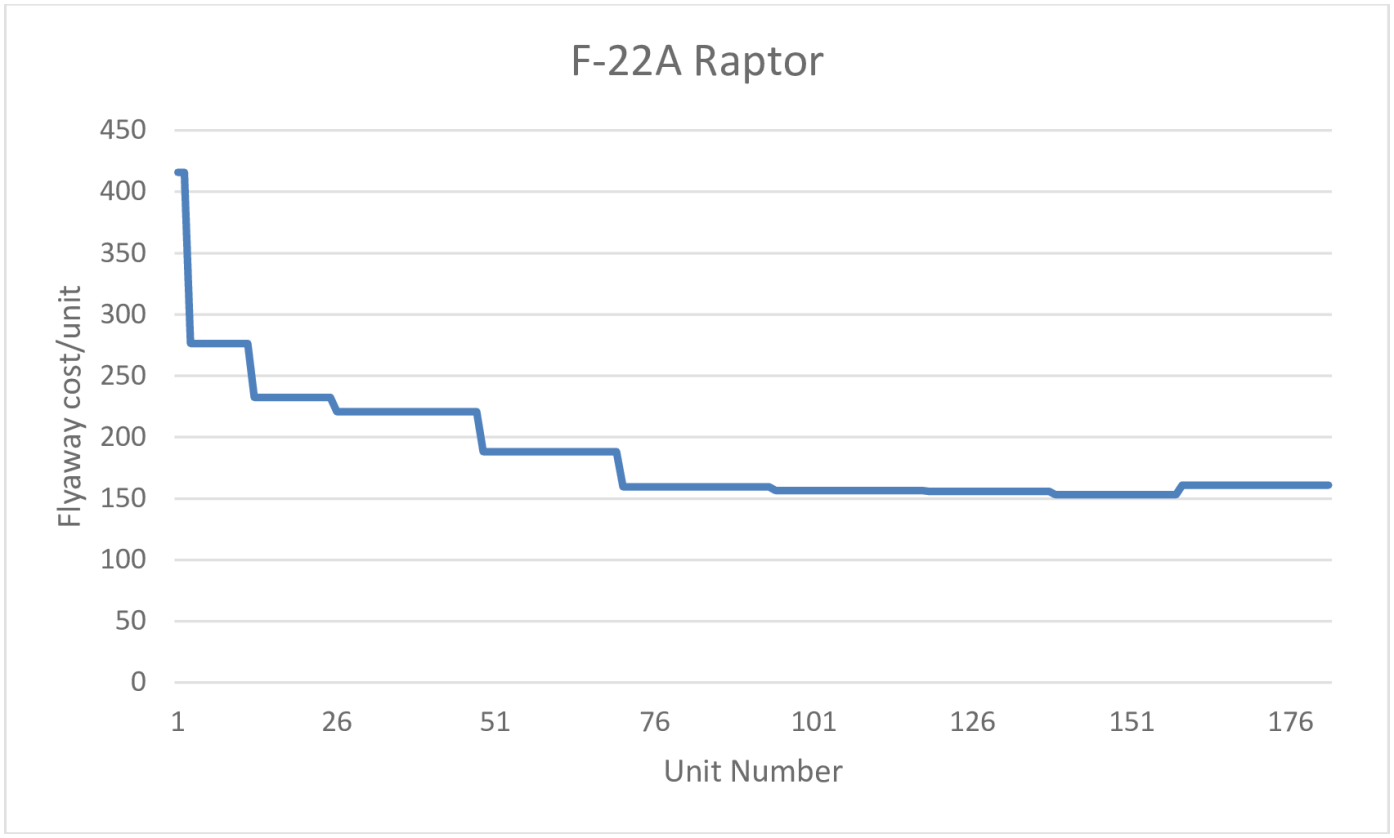
F-22A Raptor. Unit flyaway cost.

**Fig 3:**
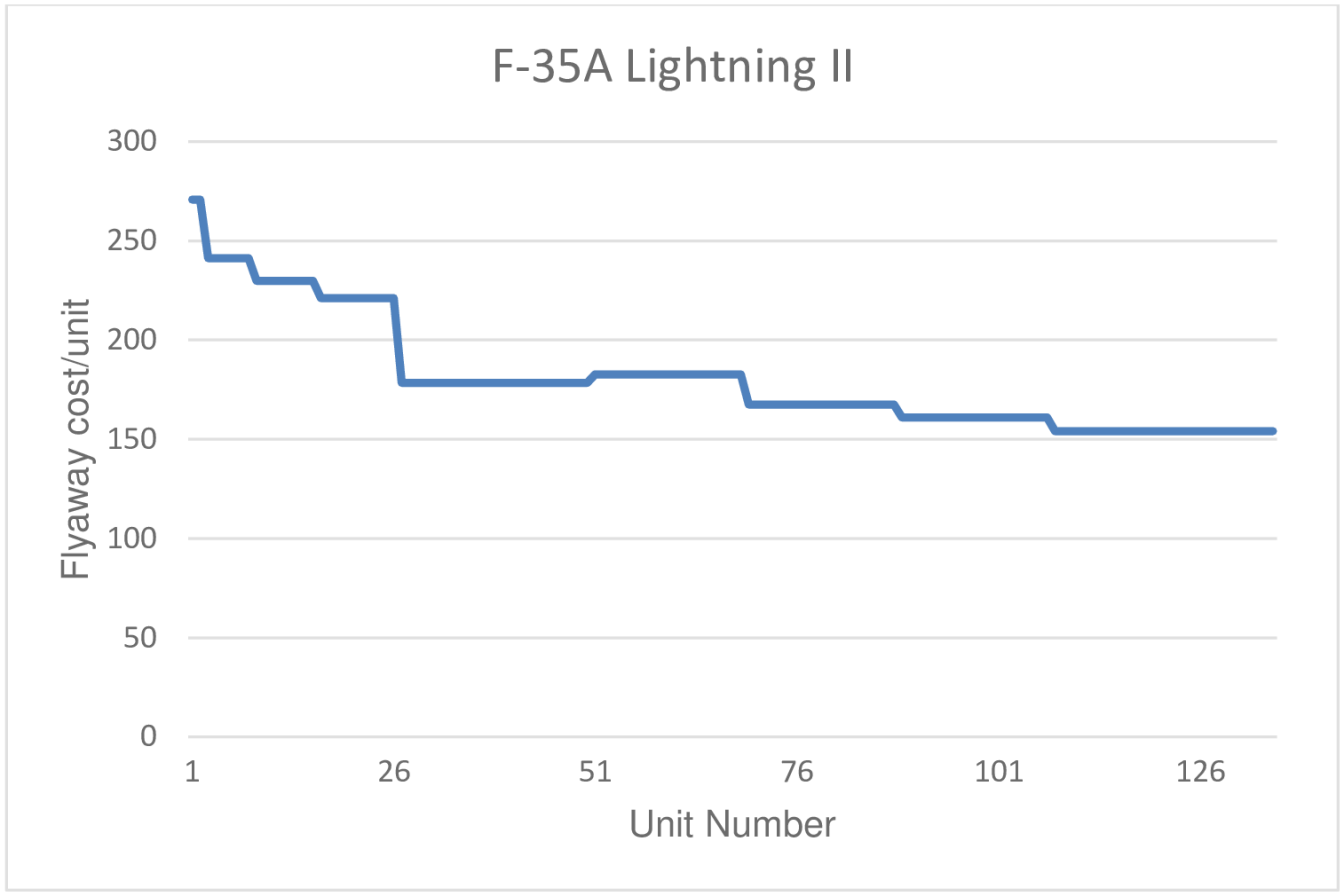
F-35A Lightning II. Unit flyaway cost.

The data for the F/A-18E/F refer to acquisitions during the fiscal period 1997-2013, with a total of 554 units purchased. Data from the F-22A correspond to purchases made by the USAF for the fiscal period 2000-2009, with a total of 182 units purchased (from a total of 195 units produced, of which 9 were test aircraft in stage I and 6 test aircraft in stage II, one airplane to replace a test aircraft lost in accident, and two EMD aircraft (Engineering and Manufacturing Development), see [59]). Finally, with respect to the F-35 Lightning II, the data correspond to version A, since the production of versions B and C is still very limited, including a total of 135 units. In this last case, data correspond to acquisitions for the fiscal period 2007-2015. Figs 1, 2 and 3 plot the flyaway cost as a function of the number of units produced for each aircraft.

Data have an annual structure and are taken directly from the budgets of the Department of Defense of the United States (DoD). The flyaway price in current dollars has been converted to constant dollars, using the DoD procurement deflator. Moreover, the output is determined in terms of a number of units acquired by the DoD. This approach is correct in the case of the F-22A Raptor and the F/A-18E/F Super Hornet, models for which all production has been acquired by the United States. However, in the case of the F-35A Lightning II, not all units produced have been acquired by the United States, as some of the units produced have been purchased by other countries. As flyaway cost refers only to units acquired by the DoD, this would implies an overestimation of learning effects for this aircraft, as actual total production had been higher than the number of units produced used in the estimation of the learning curve.

Our dataset has some particular characteristics and limitations. First, we use price data as an alternative to production cost data. In the literature we find learning curves estimated using both production cost, mainly labour cost, and product price data. Indeed, initial learning curves refer to cost, and only under certain conditions learning curves can be applied to the price of the product. This is the case when the relationship price/cost remains constant. In our case, the flyaway cost is a direct valuation of production cost. Another characteristic of the dataset is that available data refer to lots that are procured by the DoD on an annual basis. In the case of discrete production, i.e., when data refer to lots or batches, the unitary cost or price does not refer to the average unit of the lot as learning occurs continuously within the lot production. In this case, mid-unit formula must be used (see [60]). The lot or batch midpoint, *q*_*m*_, expression can be defined as:

$$\begin{array}{c} q_{m}={\left[\frac{\left(q_{f}-q_{i}+1\right)\left(1+\beta \right)}{{\left(q_{f}+0.5\right)}^{1+\beta }-{\left(q_{i}-0.5\right)}^{1+\beta }}\right]}^{-\frac{1}{\beta }} \end{array}$$ (6)

where *q*_*f*_ is the cumulated quantity at the end of the lot, *q*_*i*_ is the cumulated quantity up to the previous lot, *β* and is the learning index. The main problem for the use of the lot midpoint formula is that we need the previous value for *β*. This can be done using iterative estimation methods. Alternatively, Loerch proposed the following approximation for estimate lot midpoint [61]:

$$\begin{array}{c} q_{m}=\frac{q_{f}+q_{i}+2\sqrt{q_{f}q_{i}}}{4} \end{array}$$ (7)

All estimations have been done using raw data and lot-adjusted data using the Loerch method [61]. Nevertheless, given that the size of lots is small with a short number of units, results do not change significantly.

## Results and discussion

We begin by estimating the standard specification for the learning curve for each aircraft model, where the log of the flyaway cost is regressed against the log of cumulative production as a proxy for experience (expression (4) in logarithmic terms):

$$\begin{array}{c} \log P_{t}=\alpha +\beta \log E_{t}+\varepsilon_{t} \end{array}$$ (8)

where *α* > 0, −1 < *β* < 0, and *ε*_*t*_ is an error term. The intercept (*α* = log *a*), represents the logarithm of the cost required to produce the first unit and *β* is the learning index representing the elasticity of cost with regard to output. The slope of the learning curve is calculated as *θ* = 2^*β*^, and the learning rate is defined as 1 − *θ*. Estimation of expression (8) is done by using Ordinary Least Square. The basic specification is estimated also by using a time trend and the production rate proxied by the annual acquisition number as additional explanatory variables. A summary of the main results from the estimation is shown in Table 1.

**Table 1:** Learning curve estimation.

	(1)	(2)	(3)	(4)
**F/A-18E/F Super Hornet**				
Constant	5.700 (0.119)***	6.107 (0.123)***	6.341 (0.110)***	6.344 (0.117)***
Learning	-0.218 (0.022)***	-0.347 (0.033)***	-0.180 (0.012)***	-0.173 (0.057)***
Time	−	0.03 (0.007)***	−	-0.001 (0.011)
Production rate	−	−	-0.245 (0.035)***	-0.254 (0.076)**
*θ* (%)	85.98	78.62	88.27	88.70
*R*^2^	0.867	0.943	0.969	0.969
**F-22A Raptor**				
Constant	13.099 (0.053)***	13.109 (0.064)***	13.081(0.075)***	13.012 (0.072)***
Learning	-0.228 (0.013)***	-0.237 (0.032)***	-0.241 (0.037)***	-0.501 (0.134)***
Time	−	-0.04 (0.015)	−	0.292 (0.146)*
Production rate	−	−	-0025 (0.068)	0.064 (0.032)*
*θ* (%)	85.40	84.85	84.62	70.66
*R*^2^	0.975	0.976	0.975	0.985
**F-35A Lightning II**				
Constant	5.752 (0.061)***	5.706 (0.066)***	5.774 (0.082)***	5.765 (0.018)***
Learning	-0.135 (0.017)***	-0.064 (0.056)	-0.106 (0.071)	0.257 (0.041)***
Time	−	-0.042 (0.031)	−	-0.117 (0.012)***
Production rate	−	−	-0.049 (0.115)	-0.325 (0.038)***
*θ* (%)	91.07	−	−	−
*R*^2^	0.911	0.934	0.914	0.996

Standard error in parenthesis.

***, **, * implies significance at 1%, 5% and 10%, respectively.

Using the basic specification, where only cumulative production is used as the explanatory variable, we obtain an estimate of the learning curves of 86% for the Super Hornet; 85.4% for the Raptor; and 91% for the Lightning II. This implies that learning rates are around 14% for the first two aircraft, but around 9% for the last one. That is, for the first two models, the estimated learning rates are roughly similar, but we find evidence of a less intensive learning process in the case of the F-35A. Notice that, in spite of the fact that learning rates are roughly similar for the Super Hornet and the Raptor, the total produced quantities for these two models are very different. Estimated constants reflect the price of the first production unit. We found that the price for the first production unit is fairly similar to the Super Hornet and the Lightning II, whereas it is much higher for the Raptor, which can be explained by the fact that the F-22A was the first 5th generation aircraft to be developed and produced, representing a path-breaking in the technology of the industry.

When time is included in the regression (column 2), the estimated coefficient is only significant for the case of the Super Hornet, but with a positive value, contrary to the expected sign. This may be due to the fact that the data used are from annual batches, which cannot properly collect the assumed negative theoretical relationship between time and production costs, or to the lack of Hicks neutral technological change during the production phase. Column (3) shows the results including the production rate as an additional explanatory variable. Only in the case of the Super Hornet, the estimated parameter is significant. Finally, column (4) shows the results considering both the time and the production rate. In this case, the time trend estimated parameter is significant and with the correct sign for F-35A, while in the case of the F-22A it is significant at 10%, but with the wrong sign. However, in the case of the F-35A the estimation of the learning curve is not consistent, as the estimated learning parameter is positive, which implies a rise in production costs, once the flyaway cost has been controlled by time and acquisition rate. Note than in the case of the F-35A, learning is expected to be lower than reported, given that not all produced units are included in the data and only units procured by the DoD are considered. This means that real production is higher than the figure used in the estimation, and therefore, learning rates are overestimated for this aircraft. Notwithstanding the positive bias in the estimation of the learning index for this aircraft, the estimated learning rate is significantly lower than the estimated figures for the other two aircraft.

Next, we proceed to re-estimate the three learning curves but considering depreciation of experience. In this case, the stock of knowledge in a period of time is defined in terms of the stock of knowledge in the previous period plus the added experience between the two periods, using expression (5), so that:

$$\begin{array}{c} E_{t}=\lambda E_{t-1}+q_{t} \end{array}$$ (9)

where *E*_*t*_ is the cumulated experience until time *t*, λ = (1 − *δ*), and *q*_*t*_ is the experience gained between times *t* and *t* − 1, equivalent to the number of units produced in that period of time. The parameter λ (0 < λ < 1) measures the persistence in the stock of experience from period to period. If λ = 1 (*δ* = 0), all previously acquired experience is transmitted to the next period without suffering any depreciation. On the contrary, if λ = 0 (*δ* = 1), depreciation is total and there is no transmission of experience between periods and, therefore, learning depreciation would be complete.

In order to introduce the depreciation of experience within the learning function, we estimate, using nonlinear least squares, the following specification resulting from substituting expression (9) into expression (8):

$$\begin{array}{c} LogP_{i}=\alpha +\beta \log\left(\lambda E_{i-1}+q_{i}\right)+\gamma T_{i}+\varepsilon_{i} \end{array}$$ (10)

where *T*_*i*_ is a time trend. The estimation results are shown in Table 2, where the variable “Forgetting” refers to the estimated value of the parameter λ. Column (1) estimates the standard learning curve but including forgetting and column (2) controls for a time trend. As we can see, the results show that this parameter is not significantly different from zero for the cases of the F-22A and F-35A. This means that the depreciation of experience in the production of these units is total, on an annual basis. The estimated value for the case of the Super Hornet is 0.072, indicating that, on an annual basis, only 7.2% of the experience is maintained. With this specification, learning rates cannot be interpreted as in the standard learning curve estimation, as learning is no longer reflecting cumulative production. Learning rates are expected to be higher as cumulative experience is allowed to depreciate. Learning rates are about 30% for the Super Hornet and 15% for the Lightning II. The estimated model for the Raptor cannot be considered as the estimated learning rates with cumulated experience depreciation are lower than in the standard learning model and thus, estimation is not consistent.

**Table 2:** Learning and forgetting estimation.

	F/A-18E/F Super Hornet		F-22A Raptor		F-35A Ligtning II	
	(1)	(2)	(1)	(2)	(1)	(2)
Constant	6.481 (0.168)***	6.464 (0.188)***	13.065 (0.098)***	13.135 (0.292)***	5.965 (0.086)***	5.894 (0.721)***
Learning	-0.504 (0.046)***	-0.498 (0.054)***	-0.189 (0.072)**	-0.229 (0.192)	-0.239 (0.041)***	-0.165 (0.24)
Time	− −	-0.002 (0.011)	− −	0.012 (0.034)	− −	-0.040 (0.551)
Forgetting	0.072 (0.010)***	0.064 (0.0351)*	2.514 (5.458)	1.861 (5.120)	0.179 (0.144)	-0.019 (2.921)
*θ* (%)	70.51	70.81	87.72	−	84.73	−
*R*^2^	0.941	0.941	0.941	0.943	0.960	0.970

Standard error in parenthesis.

***, **, * implies significance at 1%, 5% and 10%, respectively.

These results are consistent with the empirical literature (see for instance, [53] and [55]) in which it is found that most knowledge is not retained by workers, even in normal production periods without stoppages in the production process, and that experience fully depreciates on a year basis. Benkard obtained a depreciation rate of 0.96, using monthly data for the L-1011 TriStar [41], which implies that 61% of the stock of experience existing at the beginning of the year survives at the end of the year, using data for a total of 250 L-1011s produced between 1970 and 1984. He argues that the low aircraft production rate is the main factor that explains the observed high forgetting. Our results seem to confirm that. Production rates are very low for both the F-22A and the F-35A, and this could explain why learning full depreciates for this two aircraft on an annual basis. Production rates for the A/F-18E/F are higher and this can explain why depreciation is less than total for this aircraft.

From the results of the estimation of the learning curves, we can draw a broad set of conclusions that are relevant in order to determine the learning process in the production of these aircraft, as well as to determine their final price. First, we obtain that the learning curve for a fifth-generation aircraft (such as the F-22A) is similar to that of a fourth-generation aircraft (such as the A/F-18E/F) despite significant technological differences between both fighters. Therefore, the higher complexity of a fifth-generation aircraft with respect to the previous generation one seems not to be a factor affecting learning rates in the assembly production phase. Moreover, the number of produced units for the A/F-18E/F is much higher than the produced units for the F-22A. The finding of a similar learning curve for the Super Hornet and the Raptor can be interpreted as evidence that the increasing technological burden on fighter aircraft does not mean a significant variation in the learning process in practice. The particular characteristics in the development and in the production of the F-35A do not allow to compare this aircraft with the other two.

Second, learning rates estimated for these two aircraft (the F-22A and the A/F-18E/F) are slightly smaller than previous estimations for older aircraft. For instance, Wright estimated a value of *β* = −0.322, which corresponds to an 80% learning curve [3], as the learning curve is calculated as *θ* = 2^*β*^. [33], [34], [40] and [56] confirmed those results for a number of military aircraft, with estimated learning curves in the range 80%-85%. Similar estimates are obtained for a number of commercial aircraft, with estimated learning curves in the range 70%-85% (examples are [29], [41] and [42]). The differences found in this study with previous estimations in the literature can be explained by the low production rates of recent fighter aircraft, as the total number of aircraft acquired by the DoD of modern fighters is much lower than that of old fighters, due to the increasing cost per unit.

Third, we find that rates of learning in the production of the F/A-18E/F Super Hornet and the 22A Raptor are much higher than those for the F-35A Lightning II. These findings have significant implications when it comes to study some elements concerning the strategy of the industry to design one single platform with different variants. Compared to the other two aircraft, the estimate for the F-35A yields different results, obtaining a learning curve of 91%, reflecting the existence of a minor learning process in practice for this model in relation to the other two. This difference may be due to certain specific characteristics associated with the production process of the F-35A. The F-35 is assembled at the Lockheed plant in Fort Worth, Texas. The distinguishing feature with the previous two models is that the assembly line produces three different versions simultaneously (A, B and C), with significant differences among them. The idea for the design of the F-35 is to use the same airframe to produce three variants with different characteristics: a vertical standard version, a takeoff and landing version and a version capable of operating from aircraft carriers. However, this simultaneous production of three versions can cause major problems regarding the acquisition of learning and depreciation. The alternation of different models on the same assembly line involves the manufacturing of a non-homogeneous product, so each version is exposed to periods when production is stopped. If the differences among versions are not very large, negative effects on learning could be of little significance. However, if there are significant differences among the three versions, this strategy could lead to a lower learning process and further depreciation of cumulated experience in production.

These results cast doubts on the development of platforms with different specifications to be produced simultaneously, especially when they show significant differences as strategy for reducing production costs and the price of acquisition in the fighter aircraft industry. In the case of the F-35, version C has larger wings, great internal fuel capacity and more robust landing gear than the other variants, while the engine of version B (with short takeoff/vertical landing capabilities) is very different from the other two variants. This makes that the simultaneous production of all three variants on the same assembly line may adversely affect the learning process in practice, slowing it down in time and even increasing depreciation. While this strategy seems appropriate to reduce fixed costs during the development stage of the aircraft, it may entail a less intensive process of learning by doing, so that unit production costs will not decrease to the expected rates. Therefore, it is not clear whether this strategy will result in a decrease in the unit price of this aircraft.

Interestingly, Kleiner, Nickelsburg and Pilarski reported the cases of the DC-9 and MD-80 aircraft production [16]. They pointed out that the DC-9 and the MD-80 shared the same fuselage cross-section, but the wing, electronics, and systems integration were different. The mixing of the two models on the same production line dramatically increases the production cost of the DC-9. Our results can be interpreted as evidence in the same direction with respect to the F-35A Lightning II.

To illustrate this question, consider the following example. Suppose that the DoD needs two types of aircraft with different technical and operational characteristics. Suppose the existence of two aerospace firms, *i* and *j*. The strategy of firm *i*, is to develop a single airframe for two differentiated (technical and operational) specifications to be produced on the same assembly line. Firm *j* develops two airframes for two different aircraft models in two different assembly lines. Assume that the total number of aircraft to be produced by both firms is the same, *N*. As a consequence, fixed costs (*FC*) in the development and research pre-production phase (RDT&E, Research, Design, Test and Evaluation costs) for firm *j* are higher than those for firm *i*. This means that fixed cost per production unit will be lower for the two aircraft models derived from the same airframe produced by firm *i* compared to the fixed cost per unit for the two aircraft models derived from different airframes by firm *j*. However, learning-by-doing in the production phase will be different. Given the learning-by-doing process, average variable cost (*AVC*) can be defined as:

$$\begin{array}{c} AVC=\frac{a\sum_{k=1}^{N}Q_{k}^{\beta }}{Q_{N}} \end{array}$$ (11)

whereas average total cost (*ATC*) would be:

$$\begin{array}{c} ATC\;=\frac{FC}{Q_{N}}+\frac{a\sum_{k=1}^{N}Q_{k}^{\beta }}{Q_{N}} \end{array}$$ (12)

For the new strategy to be the best option in reducing the final price of the aircraft, the following condition must hold:

$$\begin{array}{c} \frac{FC_{i}}{Q_{N}}+\frac{a\sum_{k=1}^{N}Q_{k}^{\beta_{i}}}{Q_{N}}<\frac{FC_{j}}{Q_{N}}+\frac{a\sum_{k=1}^{N}Q_{k}^{\beta_{j}}}{Q_{N}} \end{array}$$ (13)

Under the assumption that the number of total production units will be the same under both strategies, the above condition can be written as:

$$\begin{array}{c} \sum_{k=1}^{N}Q_{k}^{\beta_{i}-\beta_{j}}<\frac{FC_{j}-FC_{i}}{a} \end{array}$$ (14)

From that expression, we find that the optimality of each strategy depends on the following factors. First, the lower the price of the first unit (lower *a*), the better the new strategy for producing aircraft. This is because while the first unit of a particular aircraft model to be produced is very expensive, learning effect becomes more important in pricing produced units. Second, the higher the difference between fixed costs, the better the new strategy. It is clear that when fixed costs are very high and represent a significant fraction of the final price, the new industry strategy has an advantage. Third, the best strategy depends on the total number of units to be produced. As the number of total units increases, the new strategy is better. This is because when the number of units to be produced is very large, fixed costs per unit represent a small quantity. Finally, the optimal strategy depends on the difference between the learning rates (*β*_*i*_ − *β*_*j*_). Given that learning in the first strategy is lower than in the second, the greater the difference between learning rates, the worse the new strategy.

## Concluding remarks

This article studies learning curves and persistence of experience for the three most recent fighter aircraft. Learning process is particularly important in the aircraft industry, where skilled labour in the assembly process involves a high percentage of total costs and accumulated experience in the manufacturing of a particular aircraft model leads to a progressive reduction in production costs as experience increases productivity. Furthermore, the final price of an aircraft depends on the number of previously produced aircraft of the same model. In the case of military aircraft, the learning-by-doing process observed during the manufacturing process has important implications in determining the final procurement price of the equipment, given the intensity of learning acquisition in the assembly phase of this equipment. In fact, the estimation of the learning curve is one of the main tools for fixing the price of the aircraft acquired by the U.S. DoD.

Given the dramatic increases in the prices of a more advanced fighter, a recent strategy pursued by the industry to reduce the total cost of new generation fighters is the development of a unique airframe with different technical and operational specifications to meet the requirements of the DoD. This is the case of the F-35 Lightning II produced by Lockeed Martin, where three versions, A, B, and C, have been developed simultaneously, sharing a similar airframe. Whereas this strategy can be useful to reduce the costs in the pre-production phase, the question of its effects on the costs in the assembly phase still remains open. This is the main question this paper tries to answer.

We find that the learning curve of the F-22 Raptor is similar to that of the F/A-18E/F Super Hornet, reaching approximately 85%, a value slightly higher (lower learning rates) than that found in the literature for other aircraft models. In the case of the F-35A, the observed learning progress is smaller, with a learning curve of above 90%, which may be due to the special features in the production of this aircraft, using an only platform to produce three different versions, with different technical and operational specifications, simultaneously on the same assembly line. Based on these results, the design and development of a single model with very different versions do not seem to be the solution, according to the results obtained for the case of the F-35A. The simultaneous production of different versions on the same assembly line can significantly hamper the learning processes in practice, while it can facilitate the depreciation of the previously accumulated experience so that the reduction in production costs as cumulative production increases would be lower.

The estimated depreciation on experience is very high for all three models. On an annual basis, the depreciation is complete for the cases of the F-22A and F-35A. This may be due to the small number of units produced and the annual nature of the data used. In the case of the F/A-18E/F, the estimated depreciation is over 90% in annual terms. Further analyses of the learning curves for each of the variants of the F-35 may be very important in order to determine the best strategy to be followed by the aircraft industry in the development of the new fighter aircraft. Additionally, it would also be important to quantify the impact of this strategy on the expected life cycle costs of the whole program. Also, it would be of interest to study how the higher technological complexity of fighter aircraft has affected both learning and forgetting in this particular industry.

Finally, the results obtained in this paper have not only implications for the aircraft industry, but they could be of great relevance for other industries, in particular for industries facing large sunk costs in the R&D phase of new products or industries that produce a large number of varieties of a particular product. All this industries could have a trade-off between pre-production fixed costs and productivity gains in the production phase. The findings in this paper show that the correct strategy has to be chosen through the evaluation of the interactions between the production of a number of varieties of a product and the learning and forgetting processes.
